# *Cancer Reports* is transitioning to Open Access—Embracing Open Science

**DOI:** 10.1002/cnr2.1283

**Published:** 2020-08-18

**Authors:** Nidhi Bansal

**Affiliations:** ^1^ John Wiley & Sons, Inc. Hoboken, New Jersey USA

*Cancer Reports* was launched in 2018 with the philosophy—“All sound data and evidence‐driven cancer research has value and should be published and shared with the community.” Since then the journal has grown in its article output and academic impact by publishing studies that are relevant to an international audience and are of global or regional interest. With hard‐work, support, trust and scientific diligence of our authors, reviewers and editors, the journal has successfully adhered to the principles of sound science wherein manuscripts are evaluated on their scientific merit and ethical compliance as opposed to novelty or perceived impact. Recently, the journal achieved a major milestone when it was accepted for inclusion in two of the most prestigious indices, MEDLINE and the Web of Science’s Emerging Sources Citation Index (ESCI).

From its start, *Cancer Reports* has endorsed Open Science to promote scientific rigor, transparency and research reproducibility. It became one of the very few oncology journals to publish a new empirical article type, Registered  Report, that emphasizes the importance of the research question and methodology by conducting peer review before data collection thereby eliminating selective reporting and publication bias. Other important initiatives included adoption of CRediT (Contribution Roles Taxonomy)^1^ that allows a standardized description of each author's individual contribution to an article, increasing transparency around who participated in the research and the role they played. Since April 2019, all eligible articles in *Cancer Reports* publish a data availability statement to confirm the presence or absence of shared data and how it can be accessed.

Continuing its stride towards embracing Open Science, we are pleased to share the exciting news that *Cancer Reports* is transitioning to Open Access. Starting from 13^th^ August, 2020, all articles submitted and accepted for publication in *Cancer Reports* will be published Open Access under the terms of the Creative Commons Attribution (CC BY) License which permits use, distribution and reproduction in any medium, provided the original work is properly cited. All peer‐reviewed and accepted articles will be subject to an ArticleProcessing Charge (APC) to cover the cost of publishing. With this change, *Cancer Reports* joins the Wiley Open Access Portfolio. The journal will have fast production times facilitating rapid publication of articles that are immediately freely available to read, download and share. Open access confers several benefits to the authors and the research community at large. Authors retain the copyright of their article while allowing more permissive use and widest possible dissemination of their research. With increasing number of institutions and funders worldwide, encouraging and more often mandating the researchers to publish Open Access, this transition will ensure that all *Cancer Reports'* authors are compliant with their funders' Open Access policy. As a Wiley Open Access journal, articles in *Cancer Reports* will feature article discovery tools and metrics (ADMs) that will increase article discoverability, help authors connect with colleagues, and give usage metrics of individual articles.

It is important to underscore that manuscript evaluation criteria, publication ethics and peer review policy of the journal will remain unchanged and will continue to comply with rigorous research and community standards. We are truly excited as we enter the next phase of publishing in *Cancer Reports*. We look forward to the continued support of our authors, reviewers and readers and hope this change will encourage new authors to publish in *Cancer Reports*.
